# *Staphylococcus hsinchuensis* sp. nov., Isolated from Soymilk

**DOI:** 10.3390/pathogens13040343

**Published:** 2024-04-21

**Authors:** Yu-Ting Wang, Yu-Chun Lin, Yi-Huei Hsieh, Yu-Tzu Lin, Moriyuki Hamada, Chih-Chieh Chen, Jong-Shian Liou, Ai-Yun Lee, Wei-Ling Zhang, Yung-Tsung Chen, Chien-Hsun Huang

**Affiliations:** 1 Division of Research and Analysis, Food and Drug Administration, Ministry of Health and Welfare, Taipei 115021, Taiwan; 1707wyt@fda.gov.tw; 2 Taiwan Livestock Research Institute, Ministry of Agriculture, Tainan 71246, Taiwan; yclin07@mail.tfrin.gov.tw (Y.-C.L.);; 3 Fisheries Research Institute, Ministry of Agriculture, Keelung 202008, Taiwan; 4 Department of Medical Laboratory Science and Biotechnology, China Medical University, Taichung 404328, Taiwan; yutzulin@mail.cmu.edu.tw; 5 Biological Resource Center, National Institute of Technology and Evaluation (NBRC), 2-5-8 Kazusakamatari, Kisarazu 292-0818, Chiba, Japan; 6 Institute of Medical Science and Technology, National Sun Yat-sen University, Kaohsiung 80424, Taiwan; 7 Rapid Screening Research Center for Toxicology and Biomedicine, National Sun Yat-sen University, Kaohsiung 80424, Taiwan; 8 Bioresource Collection and Research Center (BCRC), Food Industry Research and Development Institute, Hsinchu 30062, Taiwanayl@firdi.org.tw (A.-Y.L.);; 9 Department of Food Science, National Taiwan Ocean University, Keelung 202301, Taiwan; ianchen619@mail.ntou.edu.tw

**Keywords:** soymilk, *Staphylococcus hsinchuensis*, new taxa, polyphasic analysis

## Abstract

A novel coagulase-negative *Staphylococcus* strain (H164^T^) was isolated from soymilk in Taiwan. Comparative sequence analysis of the 16S rRNA gene revealed that the H164^T^ strain is a member of the genus *Staphylococcus*. We used multilocus sequence analysis (MLSA) and phylogenomic analyses to demonstrate that the novel strain was closely related to *Staphylococcus gallinarum*, *Staphylococcus nepalensis*, *Staphylococcus cohnii*, and *Staphylococcus urealyuticus*. The average nucleotide identity and digital DNA-DNA hybridization values between H164^T^ and its closest relatives were <95% and <70%, respectively. The H164^T^ strain could also be distinguished from its closest relatives by the fermentation of _d_-fructose, _d_-maltose, _d_-trehalose, and _d_-mannitol, as well as by the activities of α-glucosidase and alkaline phosphatase. The major cellular fatty acids were C15:0 iso and C15:0 anteiso, and the predominant menaquinones were MK-7 and MK-8, respectively. The major cellular fatty acids and predominant menaquinones were C_15:0_ iso and C_15:0_ anteiso and MK-7 and MK-8, respectively. In conclusion, this strain represents a novel species, named *Staphylococcus hsinchuensis* sp. nov., with the type strain H164^T^ (=BCRC 81404^T^ = NBRC 116174^T^).

## 1. Introduction

Staphylococci are commonly found in the environment, in animals, and in humans. By April 2024, 72 species had received valid published names (https://lpsn.dsmz.de/search?word=staphylococcus, accessed on 20 April 2024). Coagulase-negative staphylococci (CoNS) are the major commensal microbes on human skin, and some species are recognized as opportunistic pathogens [1,2]. Currently, the dangers of staphylococci and their role in food safety have been attributed not only to *Staphylococcus aureus*, which is a well-known foodborne pathogen but also to CoNS species, owing to their ability to transfer virulence factor genes and antibiotic resistance to *S. aureus* [3].

CoNS are commonly found in raw food materials, foods containing uncooked ingredients from various sources, or foods processed by frequent manual handling. The distinct distribution of species contributes to the unique characteristics of each category [3]. Among CoNS, *Staphylococcus cohnii* has garnered attention owing to its notably high levels of multidrug resistance [4], which is typically associated with determinants harbored within mobile genetic elements [5]. The species *S. cohnii* was divided into two subspecies, *S. cohnii* subsp. *cohnii* and *S. cohnii* subsp. *urealyticus* [6], and has been reclassified as an independent species using phylogenomic analyses [7,8]. Conversely, some CoNS species are utilized as starters in meat fermentation or other food processing procedures. For example, *Staphylococcus nepalensis*, which was originally isolated from goats, has been used to enhance the aroma of fish sauce [9]. *Staphylococcus gallinarum* is generally considered nonpathogenic, and certain strains have recently emerged as promising probiotic candidates for the production of fermented coconut-based beverages and the management of kidney stone disease [10]. An increasing number of novel *Staphylococcus* species have been identified and isolated from food sources in recent years [11,12], suggesting that foodstuffs serve as reservoirs for the discovery of novel species.

Soymilk is one of the most common plant-based beverages in Asia [13], serves as a valuable source of protein, micronutrients, and phytochemicals, and has been suggested to have health advantages and health benefits that may include reducing the potential risks of cardiovascular issues and cancer [14,15,16]. Thus, soymilk has gained popularity as a preferred fermented soy product in numerous developing nations, mainly because of its comparatively low production costs and rich nutritional content, including a protein level comparable to that of cow milk, albeit with a slight difference in amino acid composition [17,18]. However, contamination may occur after processing because of poor hygiene practices. Some reports indicate that soymilk is a possible route for the transmission of foodborne pathogens, including *Citrobacter*, *Escherichia coli*, *Klebsiella*, and *Staphylococcus aureus*, thereby posing the potential risk of spreading antibiotic resistance among consumers [19,20,21].

This study aimed to analyze the microbial composition of soymilk products collected from a traditional market in Hsinchu, Taiwan. One staphylococcal strain, H164^T^, was isolated and could not be clearly assigned to any recognized species of the genus *Staphylococcus* via matrix-assisted laser desorption ionization time-of-flight mass spectrometry (MALDI-TOF MS) and 16S rRNA gene sequence analyses. However, phenotypic analysis using the VITEK 2 Compact automatic biochemical test showed 93% identity with *Staphylococcus cohnii* subsp. *cohnii*. Based on these inconsistent species identification results, we decided to conduct further genotypic and phenotypic analyses and found evidence to suggest that this strain is a novel staphylococcal species, hereafter designated *Staphylococcus hsinchuensis*.

## 2. Materials and Methods

### 2.1. Isolation of Strain H164^T^ and Culture Conditions

Soymilk products were purchased from a traditional market in Hsinchu City (approximate geographic coordinates: 24.79694° N, 120.97195° E), Taiwan, in 2017. Soymilk samples were serially diluted in a ten-fold serial dilution. Serial dilutions were plated on trypticase soy agar (TSA) plates for 48 h of aerobic incubation at 30 °C. All isolates were subjected to strain discrimination using a MALDI Microflex LT mass spectrometer (Bruker Daltonics, Bremen, Germany), as previously described [22]. However, strain H164^T^ could not be reliably identified. Strain H164^T^ and its phylogenetically closest species, including *S. gallinarum* (BCRC 13913^T^), *S. nepalensis* (CCUG 48991^T^), *S. cohnii* (ATCC 29974^T^), and *S. urealyuticus* (ATCC 49330^T^), were routinely cultured on TSA at 30 °C for further taxonomic characterization, and the strains were then preserved in 10% glycerol at −80 °C.

### 2.2. DNA Extraction and Gene Sequence Comparison

Bacterial DNA was extracted and purified using the QIAGEN DNeasy Blood & Tissue Kit (QIAGEN, Hilden, Germany). The 16S rRNA gene and six housekeeping genes (*dnaJ*, *gap*, *hsp60*, *rpoB*, *sodA*, and *tuf*) were amplified and sequenced using previously described primer pairs [23,24,25,26,27,28,29,30]. BLAST analysis of the target genes was performed using the EzBioCloud server (https://www.ezbiocloud.net/, accessed on 20 April 2024) and the GenBank database (www.ncbi.nlm.nih.gov/BLAST/, accessed on 20 April 2024), respectively.

### 2.3. Phylogenetic Analysis

Clustal_x version 2.1 software was used for aligning sequences [31]. MEGA (v. 11) software was used for phylogenetic tree reconstruction [32] based on sequences from the novel strain H164^T^, its closely related strains, a roughly 1450 bp segment of the 16S rRNA gene, and nearly 9600 bp of the concatenated sequences of the six housekeeping genes. Neighbor-joining (NJ) [33], minimum-evolution (ME) [34], and Kimura two-parameter models were used for tree reconstruction. Bootstrapping analysis with 1000 replicates was performed to determine the statistical reliability of the trees [35].

### 2.4. Genomic Analysis

Bacterial DNA was extracted using QIAGEN Genomic Tip columns (QIAGEN, Hilden, Germany), sequenced on a PacBio Sequel device platform (Pacific Bioscience, Menlo Park, CA, USA) with a 10-kb size-selected insert library via the continuous long read (CLR) sequencing mode. A total of >400,000 reads with a base total of >3000 Mbp total bases and a mean read length of >7 kb were produced. Highly accurate Hifi reads generated from CLR were analyzed using SMRT Analysis version 10.2 (Pacific Bioscience, Menlo Park, CA, USA). De novo assembly was performed using Microbial Assembly, SMRT Analysis version 10.2, and Flye. Differences between the assembly sequences from the Microbial Assembly and Flye were confirmed using Sanger sequencing. The complete genome sequence was deposited in GenBank CP128355 under BioProject PRJNA979948. The NCBI Prokaryotic Genome Annotation Pipeline (PGAP) was used to annotate the genome [36]. Overall genome-related index methods were used to calculate the average nucleotide identity (ANI) using the online tool ANI Calculator [37]. The digital DNA–DNA hybridization (dDDH) was calculated by formula 2 of the Genome-to-Genome Distance Calculator version 3.0 [38]. An up-to-date bacterial core gene (UBCG) pipeline [39] was used to construct a phylogenomic tree. The NJ dendrogram and hierarchical clustering based on gene content (presence or absence) were generated using the ComplexHeatmap R package [40]. The eggNOG 4.5 and carbohydrate-active enzyme (CAZy) databases were used for functional assignment [41,42]. Putative biosynthetic gene clusters were predicted using AntiSMASH software (v. 6.0) [43]. Antimicrobial resistance genes were searched for in the genome of strain H164^T^ based on the Comprehensive Antibiotic Resistance Database (CARD) Variants v4.0.0 [44], ResFinder v4.0 [45], and AMRFinderPlus v3.10.42 [46] using ProbioMinServer [47].

### 2.5. Colony Morphology and Growth Requirements

The novel strain H164^T^ was aerobically cultivated on tryptic soy agar (TSA) (BD, Heidelberg, Germany) at 37 °C overnight unless otherwise stated. Gram staining was performed using a Gram staining kit (Difco Laboratories, Detroit, MI, USA), according to the manufacturer’s instructions. The aerobic requirements were estimated using Brewer’s thioglycolate medium (Merck, Darmstadt, Germany) [48]. Growth at 15, 30, 37, 42, and 45 °C [49], in the presence of 0, 5, 7.5, 10, and 15% NaCl [50] at pH levels of 4.0–11.0 (at 1.0 pH unit intervals) was tested on P agar [51]. Colony morphologies were observed on TSA, TSA supplemented with 5% sheep blood, P agar, and Baird-Parker agar (Creative Life Science Co., Ltd., New Taipei City, Taiwan).

### 2.6. Phenotypic Characterization and Metabolic Profiling

Catalase activity was tested by API^®^ ID color catalase (Biomérieux, Marcy l’Etoile, France). Coagulase activity was determined by BD BBL™ Rabbit Coagulase Plasma (BD, Sparks, MD, USA) (35 °C, overnight). DNase activity was evaluated using DNase agar containing toluidine blue (Merck, Darmstadt, Germany). Oxidase activity was determined using BD BBL™ Oxidase Reagent Droppers (BD, Sparks, MD, USA). Motility was evaluated using BBL™ Motility Test Medium (BD, Sparks, MD, USA). The urease activity was determined using a urease agar slant (Creative Life Science Co., Ltd., New Taipei City, Taiwan). The ability of the cells to utilize various sources of carbon and their enzyme activity were evaluated using the API bacterial identification systems APIStaph, ID32, and the API Coryne test (Biomérieux, Marcy l’Etoile, France), following the manufacturer’s instructions.

### 2.7. Chemotaxonomic Characterization

MALDI-TOF MS was performed for whole-cell protein analysis in accordance with a method described previously [22]. Dendrogram clustering was constructed with a setting of 200 (distance measure: correlation; linkage: average; score-oriented) using MALDI Biotyper version 3.1 (Bruker Daltonics). Biomass for analysis of whole-cell fatty acids, polar lipids, and isoprenoid quinone was obtained by culturing strain H164^T^ in TSB for 2 d at 30 °C. Fatty acids in whole cells were extracted, saponified, and esterified, followed by automated GC analysis of the fatty acid methyl esters (FAMEs) according to the procedures required by the Sherlock Microbial Identification System (MIDI) [52] using Sherlock phospholipid fatty acids (PLFA) analysis software version 6.2B. The molecular species and concentrations of isoprenoid quinones were determined as described by Hamada et al. [53].

### 2.8. Antimicrobial Susceptibility Testing

Susceptibility to cefoxitin, benzylpenicillin, oxacillin, gentamicin, ciprofloxacin, levofloxacin, erythromycin, clindamycin, linezolid, daptomycin, vancomycin, and tetracycline was determined using a VITEK 2 Compact (Biomérieux, Marcy l’ Etoile, France) and AST card P638. BD BBL™ Sensi-Disc™ susceptibility test discs (BD, Sparks, MD, USA) were used to evaluate the susceptibility to novobiocin (5 μg), fusidic acid (10 μg), moxifloxacin (5 μg), minocycline (30 μg), rifampin (5 μg), and chloramphenicol (30 μg) using the disc diffusion method, and the breakpoints used to indicate antibiotic resistance were according to the guidelines of the Clinical and Laboratory Standards Institute (CLSI) (for all antimicrobial agents tested except fusidic acid) and the European Committee on Antimicrobial Susceptibility Testing (EUCAST) (for fusidic acid) [3]. *S. aureus* ATCC 29213 was used as the reference strain. The minimum inhibitory concentrations (MICs) of benzylpenicillin and ampicillin were determined by the E test (Biomérieux, Marcy l’Etoile, France).

## 3. Result and Discussion

**Figure 1:**
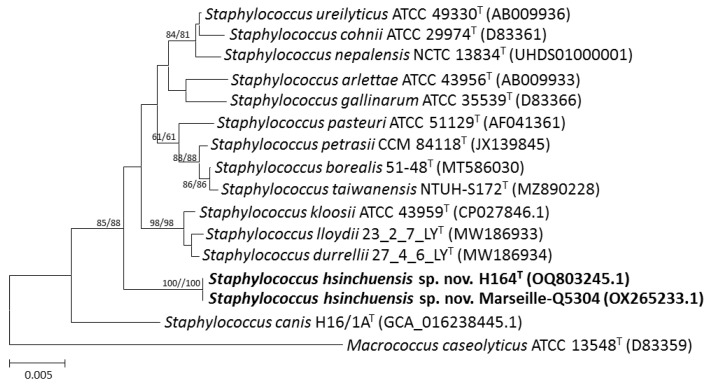
Phylogenetic tree based on 16S rRNA gene sequences showing the relationship of *Staphylococcus hsinchuensis* sp. nov. H164^T^ with strains of closely related species. The tree was constructed by the neighbor-joining and minimum evolution methods based on a comparison of approximately 1450 bp, and *Macrococcus caseolyticus* ATCC 13548^T^ was used as the outgroup. Bootstrap values (>60%) based on 1000 replicates are shown at branch nodes. Bar, 0.5% sequence divergence.

We used MALDI-TOF MS to isolate the strains from the soymilk products, and these strains were identified at the species level with log-score values higher than 2.0, except for one strain, H164^T^, which was considered unreliable, as it yielded a log score of <1.7. Comparative analysis of the 16S rRNA gene sequences revealed that the type strains *S*. *urealyticus* ATCC 49330^T^ (98.4% similarity), *Staphylococcus canis* H16/1A^T^ (98.4%), *Staphylococcus lloydii* 23_2_7_LY^T^ (98.2%), *Staphylococcus kloosii* ATCC 43959^T^ (98.2%), *S. cohnii* ATCC 29974^T^ (98.2%), *Staphylococcus petrasii* CCM 84118^T^ (98.2%), *Staphylococcus durrellii* 27_4_6_LY^T^ (98.1%), *Staphylococcus arlettae* ATCC 43956^T^ (98.1%), and *S. gallinarum* ATCC 35539^T^ (98.0%) were the closest neighbors to the novel strain H164^T^. These values were lower than the suggested cutoff value for species delineation (98.7%) [54]. Strain H164^T^ was also closely related to *Staphylococcus* sp. strain Marseille-Q5304 (NCBI: txid 2942200, an unclassified *Staphylococcus* strain isolated from a clinical sample, reported by IHU–Méditerranée Infection in 2022), with 100% 16S rRNA gene sequence similarity, followed by several uncultured environmental bacterial clones (approximately 98.9–100%). Phylogenetic analysis based on the 16S rRNA gene sequences showed that the novel strain H164^T^ belonged to the genus *Staphylococcus* (Figure 1).

**Figure 2:**
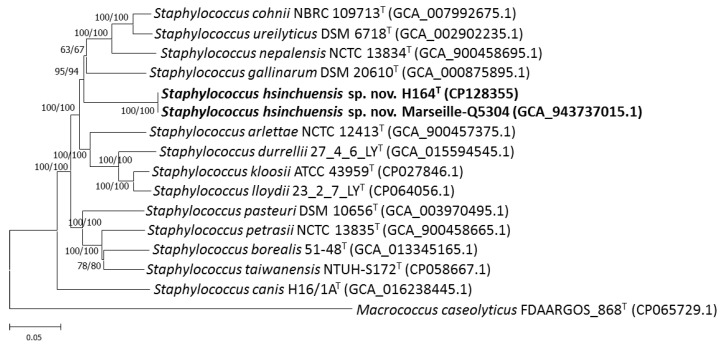
Phylogenetic tree based on the concatenated housekeeping gene sequences (*dnaJ*, *gap*, *hsp60*, *rpoB*, *sodA*, and *tuf*) showing the relationship of *Staphylococcus hsinchuensis* sp. nov. H164^T^ with strains of closely related species. The tree was constructed by the neighbor-joining and minimum evolution methods based on a comparison of approximately 9600 bp, and *Macrococcus caseolyticus* FDAARGOS_868^T^ was used as an outgroup. Bootstrap values based on 1000 replicates are shown at branch nodes. Bar, 5% sequence divergence.

Multilocus sequence analysis (MLSA) based on housekeeping gene sequences provides greater discriminative power than the 16S rRNA gene for identifying and classifying the genus *Staphylococcus* [55]. Lin et al. [56] successfully used an MLSA scheme based on six housekeeping genes (*dnaJ*, *gap*, *hsp60*, *rpoB*, *sodA*, and *tuf*) to discriminate between a novel species of *Staphylococcus hsinchuensis* and its close relatives. Hence, these genes could act as taxonomic markers and were used to characterize the novel strain. The similarity levels of *dnaJ*, *gap*, *hsp60*, *rpoB*, *sodA*, *tuf*, and the concatenated gene sequences between strains H164^T^ and Marseille-Q5304 ranged from 99.9 to 100%. The similarity values of housekeeping gene sequences shared by the strains H164^T^, *S. gallinarum* DSM 20610^T^, *S. nepalensis* CCM 7045^T^, *S. urealyticus* DSM 6718^T^, *S. cohnii* NCTC 11041^T^, *S. arlettae* NCTC 12413^T^, *S. durrelii* NCTC 14454^T^, *S. kloosii* NCTC 12415^T^, and *S. lloydii* NCTC 14453^T^ ranged from 80.2 to 93.6% (Table 1), which were clearly lower than the cutoff criteria for species differentiation [18,23,24,25,26,29]. A phylogenetic tree based on the sequences of six concatenated housekeeping genes (*dnaJ*, *gap*, *hsp60*, *rpoB*, *sodA*, and *tuf*), which was constructed by the neighbor-joining and minimum evolution methods, showed that the novel strains H164^T^ and Marseille-Q5304 formed an independent cluster that was clearly separated from *S. gallinarum*, *S. nepalensis*, *S. urealyticus*, and *S. cohnii* (Figure 2), indicating that these two strains could be novel species within the genus *Staphylococcus*.

**Figure 3:**
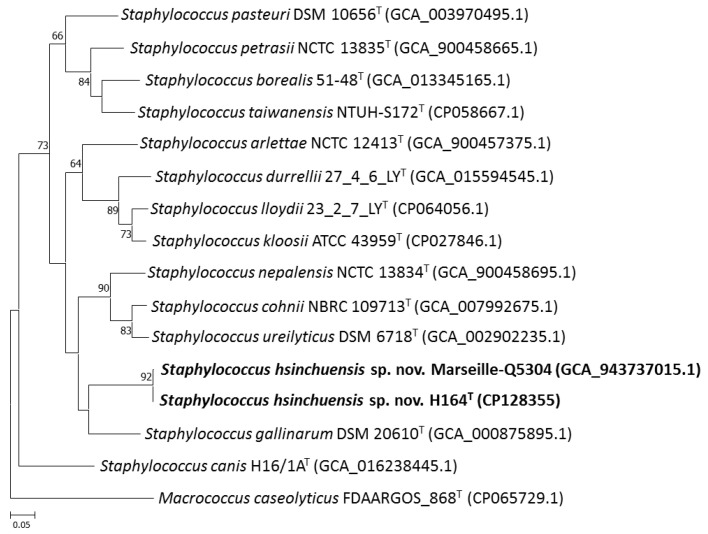
A UBCG tree based on 92 bacterial core genes of *Staphylococcus hsinchuensis* sp. nov. H164^T^ and the type strains of closely related species. Bootstrap values greater than 60% are shown at each node, and *Macrococcus caseolyticus* FDAARGOS_868^T^ was used as an outgroup. Bootstrap values based on 1000 replicates are shown at branch nodes. Bar, 5% sequence divergence.

Currently, overall genome-related indices, such as ANI and dDDH, and phylogenomic tree analyses are representative data for estimating evolutionary distances and defining prokaryotic taxa not only at the genus but also at the species level [57]. The completely assembled genome of strain H164^T^ comprised one circular chromosome (2196954 bp) and one circular plasmid (81646 bp). The G+C content of the complete strain H164^T^ genome was 33.82 mol%. Between H164^T^ and its closely related species, the ANI and dDDH varied from 74.9% to 92.0% and 20.4% to 46.4%, respectively (Table 2), which were lower than the generally accepted cutoffs of 95–96% and 70%, respectively, for prokaryotic species. The phylogenetic trees obtained using UBCG and TYGS showed that the novel strains formed an independent cluster and were most closely related to *S. gallinarum* DSM 20610^T^ (Figure 3, Supplementary Figure S1), which was consistent with the results of the heatmap and NJ dendrogram analysis (Supplementary Figure S2). These results confirm that strains H164^T^ and Marseille-Q5304 represent novel species of the genus *Staphylococcus*.

A total of 2211 genes from strain H164^T^ were assigned to 21 functional categories (Supplementary Figure S3). The most common categories among these functional groups belonged to Clusters E (amino acid transport and metabolism; 176 genes), J (translation, ribosomal structure, and biogenesis; 168 genes), and K (transcription; 167 genes). Among the identified CAZy families, strain H164^T^ contained fourteen glycosyltransferases, nine glycoside hydrolases, seven carbohydrate-binding modules, one carbohydrate esterase, and one polysaccharide lyase. H164^T^ appeared to produce putative secondary metabolite gene clusters such as cyclic lactone autoinducer, lanthipeptide class II, RiPP-like, siderophore, and terpene biosynthetic clusters.

The cells of strain H164^T^ were Gram-positive cocci in clusters, as observed under an optical microscope. The strains were nonmotile, as confirmed by motility test media, and facultatively anaerobic, as determined by Brewer thioglycollate media. After 24 h of aerobic incubation, the H164^T^ strain displayed smooth, circular, slightly convex colonies reaching 0.8 mm in diameter on TSA, nonhemolytic colonies on TSA supplemented with 5% defibrinated sheep blood, and no pigmentation on P agar. On Baird-Parker agar, a selective medium for isolating *Staphylococcus* species, strain H164^T^ displayed black colonies, and no clear zones around the colonies or opaque zones of precipitation were observed, indicating that strain H164^T^ could reduce tellurite, though it negatively affected lecithinase production and lipase activity. The H164^T^ strain was grown at 30 to 42 °C but not at 15 °C or 45 °C, was tolerant to NaCl up to 10% (survival but weak in the presence of 15% NaCl), and was tolerant in a pH of 5–9 (survival but weak in the presence of pH 11).

Strain H164^T^ was oxidase-, DNAse-, urease- and coagulase-negative and catalase-positive. Table 3 lists the phenotypic characteristics that can be used to distinguish this novel strain from its close relatives, including negative fermentation of _d_-fructose, _d_-maltose, _d_-trehalose, _d_-mannitol, and negative reactions obtained from α-glucosidase, and alkaline phosphatase. Cluster analysis of the MALDI-TOF MS spectra in the 2000–12,000 *m*/*z* region of *Staphylococcus* strains revealed an unambiguous grouping of five distinct clusters, each defined by known species and our novel taxon (Supplementary Figure S4). Fatty acid analysis revealed that strain H164^T^ contained anteiso-C_15:0_ and iso-C_15:0_ as the major fatty acids (>10% total fatty acids) and could be differentiated from the closest *Staphylococcus* species based on C18:0 and iso-C16:0 (Table 4). The predominant isoprenoid quinones in strain H164^T^ were MK-7 and MK-8, and MK-6 was detected as a minor component (65:30:5).

The determinant of penicillin resistance, *blaZ*, was found in the 81,464 bp plasmid carried by strain H164^T^. The entire *blaZ gene*, an 846 bp gene under the control of two adjacent regulatory genes, the antirepressor *blaR1* and the repressor *blaI* [58], encodes a β-lactamase that inactivates penicillin by hydrolyzing the β-lactam ring [59]. Clusters of *blaZ*, *blaR1*, and *blaI* were found in the plasmid carried by strain H164^T^. *BlaZ*, which is carried by plasmids and transferred among staphylococci, has been suggested to be involved in the major mechanism underlying penicillin resistance [60]. However, an antimicrobial susceptibility test using the VITEK 2 AST card indicated that strain H164^T^ was susceptible to benzylpenicillin. The E test was conducted to determine the MICs of benzylpenicillin. Strain H164^T^ displayed weak penicillin resistance, with an MIC of 0.25 μg/mL, which was the lower limit defined as penicillin resistance. In addition, we evaluated the susceptibility to other antimicrobial agents commonly used clinically for staphylococcal infections. The H164^T^ strain was susceptible to all antimicrobial agents tested except novobiocin, including cefoxitin, benzylpenicillin, oxacillin, gentamicin, ciprofloxacin, levofloxacin, erythromycin, clindamycin, linezolid, daptomycin, vancomycin, tetracycline, fusidic acid, moxifloxacin, minocycline, rifampin, and chloramphenicol.

## 4. Conclusions

Accordingly, the results obtained from phenotypic characterization, genomic and phylogenetic analyses, and chemotaxonomic analyses demonstrated that the novel strain H164^T^ represents a single novel species within the genus *Staphylococcus*, for which the name *Staphylococcus hsinchuensis* sp. nov. is proposed, with strain H164^T^ (=BCRC 81404^T^ = NBRC 116174^T^) as the type strain.

**Description of** ***Staphylococcus*** ***hsinchuensis* sp. nov.**

*Staphylococcus hsinchuensis* (hsin.chu.en’sis. N.L. masc. adj. *hsinchuensis* of Hsinchu, from where the type strain was isolated).

The cells are Gram-positive, facultatively anaerobic, non-motile, and coagulase-negative cocci. Colonies are approximately 0.8 mm in diameter, smooth, slightly convex, and circular on TSA, black without a clear zone around them and without an opaque zone of precipitation on Baird-Parker agar, non-pigmented on TSA, and non-hemolytic after 24 h of incubation at 37 °C under atmospheric conditions. It is catalase-positive, oxidase-negative, DNase-negative, and urease-negative. It is able to grow in 0–10% NaCl at a pH of 5.0–10.0 at 30–42 °C, whereas it slightly grows in 15% NaCl or at pH 11.0. In API bacterial identification systems, acid production is detected from _d_-glucose and sucrose; however, not from _d_-fructose, _l_-arabinose, _d_-ribose, _d_-mannose, _d_-xylose, _d_-lactose, _d_-turanose, _d_-cellobiose, _d_-maltose, _d_-trehalose, _d_-melibiose, _d_-raffinose, glycogen, *N*-acetyl-glucosamine, methyl α-_d_-glucopyranoside, _d_-mannitol, and xylitol. Positive reactions are obtained from pyrazinamidase, though not from β-glucosidase, *N*-acetyl-β-glucosaminidase, α-glucosidase, β-galactosidase, β-glucuronidase, alkaline phosphatase, arginine arylamidase, pyrrolidonyl arylamidase, ornithine decarboxylase, arginine dihydrolase, reduction of nitrates, acetoin production, and hydrolysis of gelatin. The major cellular fatty acids and isoprenoid quinones are C18:0 and iso-C16:0 and MK-7 and MK-8, respectively. It is resistant to novobiocin; however, it is susceptible to cefoxitin, benzylpenicillin, ampicillin, oxacillin, gentamicin, ciprofloxacin, levofloxacin, erythromycin, clindamycin, linezolid, daptomycin, vancomycin, tetracycline, fusidic acid, moxifloxacin, minocycline, rifampin, and chloramphenicol. The type strain H164^T^ (=BCRC 81404^T^ = NBRC 116174^T^) was isolated from soymilk. The genome size of the type strain was 2,278,600 bp, and it had a DNA G + C content of 33.82 mol%. The GenBank accession numbers are OQ803245.1 (16S rRNA gene) and CP128355 (chromosome).

## Figures and Tables

**Table 1:** Sequence similarity of the *Staphylococcus hsinchuensis* sp. nov. H164^T^ and its closely related type strains.

Type Strain	Sequence Similarity (%) with *Staphylococcus hsinchuensis* sp. nov. H164^T^						
	*dnaJ* (88.8) ^a^	*gap* (96.0)	*hsp60* (93.0)	*rpoB* (93.6)	*sodA* (97.0)	*tuf* (98.0)	MLSA ^b^
*Staphylococcus gallinarum* DSM 20610^T^	82.1	87.3	84.6	89.2	90.8	93.6	88.0
*Staphylococcus nepalensis* CCM 7045^T^	82.5	87.3	83.4	87.7	88.2	92.0	87.0
*Staphylococcus urealyticus* DSM 6718^T^	80.3	84.8	83.0	86.4	87.0	93.3	87.2
*Staphylococcus cohnii* NCTC 11041^T^	81.8	87.4	83.6	88.9	89.3	91.7	87.4
*Staphylococcus arlettae* NCTC 12413^T^	82.2	86.6	83.7	87.7	88.3	92.3	86.9
*Staphylococcus durrelii* NCTC 14454^T^	81.0	87.2	82.6	86.8	89.0	90.4	86.1
*Staphylococcus kloosii* NCTC 12415^T^	80.2	87.5	83.1	87.6	89.2	91.7	86.7
*Staphylococcus lloydii* NCTC 14453^T^	80.3	87.2	82.9	87.8	89.0	91.8	86.7

^a^ Similarity cutoff for species differentiation. ^b^ MLSA: six concatenated housekeeping gene sequences (*dnaJ*, *gap*, *hsp60*, *rpoB*, *sodA*, and *tuf*) were used.

**Table 2:** Average nucleotide identity (ANI) and dDDH prediction values (%) between the strain H164^T^ and its closely related species.

Species	Strain	Accession No.	1	2	3	4	5	6	7	8	9
1. *Staphylococcus hsinchuensis*	H164^T^	CP128355	100.0	21.5	21.6	22.0	21.9	21.5	21.1	20.7	20.4
2. *Staphylococcus gallinarum*	DSM 20610^T^	GCA_000875895.1	76.4	100.0	21.4	21.1	21.4	21.2	20.8	20.4	20.7
3. *Staphylococcus nepalensis*	CCM 7045^T^	GCA_900458695.1	76.1	76.9	100.0	26.7	27.1	21.6	20.8	20.9	20.9
4. *Staphylococcus urealyticus*	DSM 6718^T^	GCA_002902235.1	76.3	76.7	83.1	100.0	46.4	21.6	20.5	21.1	20.9
5. *Staphylococcus cohnii*	NCTC 11041^T^	GCA_007992675.1	76.4	77.1	83.2	92.0	100.0	21.6	20.7	20.9	20.9
6. *Staphylococcus arlettae*	NCTC 12413^T^	GCA_900457375.1	74.9	76.0	76.0	75.8	76.0	100.0	21.5	21.5	21.7
7. *Staphylococcus durrelii*	NCTC 14454^T^	GCA_015594545.1	75.1	75.4	75.8	75.4	75.7	77.0	100.0	31.0	30.2
8. *Staphylococcus kloosii*	NCTC 12415^T^	CP027846.1	75.1	75.4	75.7	75.8	75.8	77.0	86.3	100.0	42.9
9. *Staphylococcus lloydii*	NCTC 14453^T^	CP064056.1	75.2	75.6	75.7	76.0	75.9	77.2	85.8	91.1	100

The numbers shown on the upper right are the dDDH values (%) and the ANI values (%) are shown on the lower left.

**Table 3:** Differential biochemical characteristics of *Staphylococcus hsinchuensis* sp. nov. H164^T^ and the closest related species of the genus *Staphylococcus* strains: 1. *Staphylococcus hsinchuensis* H164^T^; 2. *Staphylococcus cohnii* NCTC 11041^T^; 3. *Staphylococcus urealyticus* DSM 6178^T^; 4. *Staphylococcus gallinarum* DSM 20610^T^; and 5. *Staphylococcus nepalensis* CCM 7045^T^. +, positive; −, negative.

Biochemical Test	1	2	3	4	5
Acid production:					
_d_-Fructose	−	+	+	+	+
_l_-Arabinose	−	−	−	+	+
_d_-Mannose	−	−	+	+	+
_d_-Xylose	−	−	−	+	+
Sucrose	+	−	−	+	+
_d_-Lactose	−	−	+	+	+
_d_-Turanose	−	−	−	+	−
_d_-Cellobiose	−	−	−	+	−
_d_-Maltose	−	+	+	+	+
_d_-Trehalose	−	+	+	+	+
_d_-Melibiose	−	−	−	+	−
_d_-Raffinose	−	−	−	+	−
*N*-Acetyl-glucosamine	−	−	+	+	+
_d_-Mannitol	−	+	+	+	+
Xylitol	−	+	+	−	+
Nitrate reduction	−	−	−	+	+
Acetoin production	−	−	+	−	−
β-Glucosidase	−	−	−	+	+
Urease	−	−	+	+	+
*N*-Acetyl-β-glucosaminidase	−	−	−	−	+
α-Glucosidase	−	+	+	+	+
β-Galactosidase	−	−	+	+	+
β-Glucuronidase	−	−	+	+	+
Alkaline phosphatase	−	+	+	+	+
Pyrrolidonyl arylamidase	−	−	+	+	+

**Table 4:** Cellular fatty acid compositions (%) of *Staphylococcus hsinchuensis* sp. nov. and its phylogenetically closely related species. Strains: 1, *Staphylococcus hsinchuensis* sp. nov. H164^T^; 2, *Staphylococcus ureilyticus* ATCC49330^T^; 3, *Staphylococcus cohnii* ATCC 29974^T^; 4, *Staphylococcus nepalensis* CCUG 48991^T^; and 5, *Staphylococcus gallinarum* BCRC 13913^T^. Values are percentages of total fatty acids. Fatty acids present at >10% are indicated in bold. TR, trace amount.

Fatty Acid	1	2	3	4	5
Saturated					
C_14:0_	3.72	3.17	3.44	2.09	1.81
C_16:0_	TR	TR	TR	TR	TR
C_18:0_	8.34	3.53	4.09	2.07	1.69
C_20:0_	3.32	2.32	1.08	TR	1.23
Branched-chain fatty acids					
iso-C_13:0_	TR	TR	1.37	TR	TR
iso-C_14:0_	3.11	2.17	2.44	TR	1.48
iso-C_15:0_	**16.85**	**16.45**	**17.20**	**17.63**	**26.59**
iso-C_16:0_	TR	2.76	1.20	1.07	2.38
iso-C_17:0_	5.64	4.99	**11.15**	5.73	7.08
iso-C_19:0_	3.34	1.22	2.28	TR	TR
anteiso-C_15:0_	**44.77**	**49.33**	**47.03**	**53.16**	**45.63**
anteiso-C_17:0_	4.92	**11.36**	6.17	**14.51**	8.34
anteiso-C_19:0_	1.63	TR	TR	TR	1.23

## Data Availability

Data are contained within the article and Supplementary Materials.

## Supplementary Materials

The following supporting information can be downloaded at: https://www.mdpi.com/article/10.3390/pathogens13040343/s1, Figure S1. Phylogenomic tree based on TYGS results showing the relationship between *Staphylococcus hsinchuensis* sp. nov. and its phylogenetically related species. The tree was inferred with FastME 2.1.6.1 from GBDP distances calculated from genome sequences. The branch lengths are scaled in terms of GBDP distance formula *d*_5_. The tree was rooted at the midpoint. Figure S2. Heat map and NJ dendrogram of the analyzed eight *Staphylococcus* strains based on the presence or absence of genes. Figure S3. Results of an eggNOG functional category analysis of strain H164^T^. The major two parts of 2114 COG categories in strains H164^T^, are E (Amino acid transport and metabolism), J (Translation, ribosomal structure, and biogenesis), and K (Transcription). Figure S4. Dendrogram showing the clustering of the *Staphylococcus* strains based on MALDI-TOF MS analysis.
