# Supplementary material for: Staphylococcus hsinchuensis sp. nov., Isolated from Soymilk
Source: Pathogens. 2024 Apr 21;13(4):343. doi: 10.3390/pathogens13040343 (PMC11055063; doi:10.3390/pathogens13040343)
Supplement: Supplementary file 1 [file pathogens-13-00343-s001.zip › pathogens-2978369-supplementary.pdf]

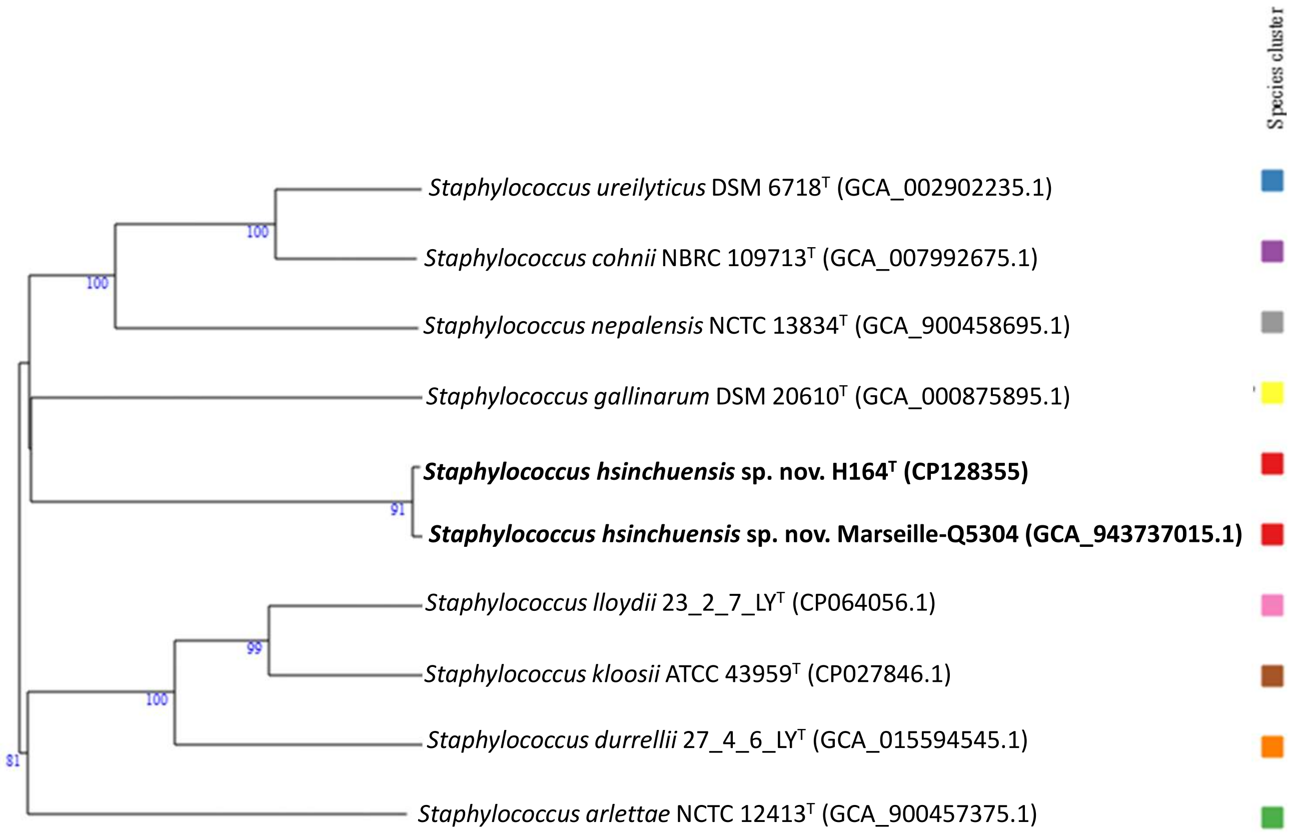

Supplementary Figure. S1. Phylogenomic tree based on TYGS results showing the relationship between *Staphylococcus hsinchuensis* sp. nov. and its phylogenetically related species. The tree was inferred with FastME 2.1.6.1 from GBDP distances calculated from genome sequences. The branch lengths are scaled in terms of GBDP distance formula d_5. The tree was rooted at the midpoint.

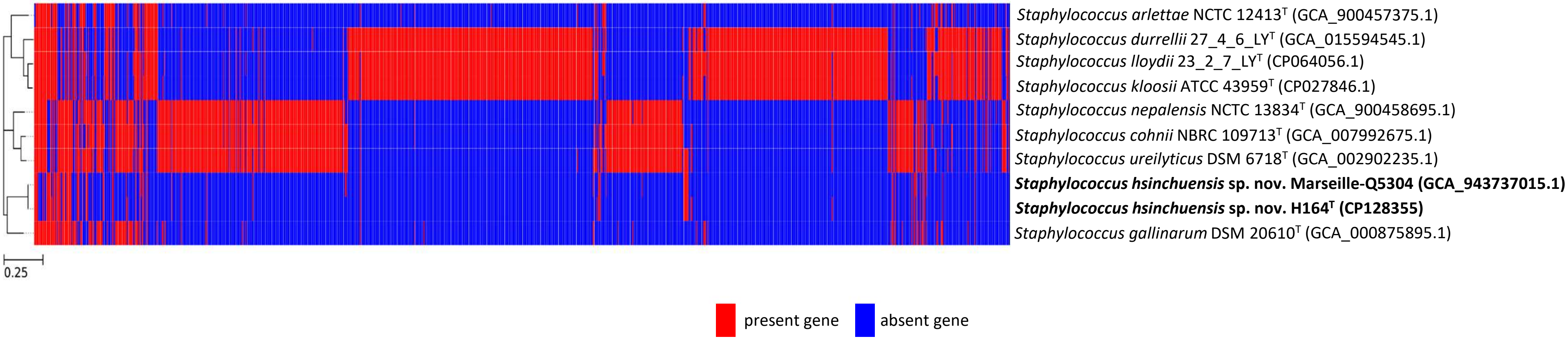

Supplementary Figure. S2. Heat map and NJ dendrogram of the analyzed eight *Staphylococcus* strains based on the presence or absence of genes.

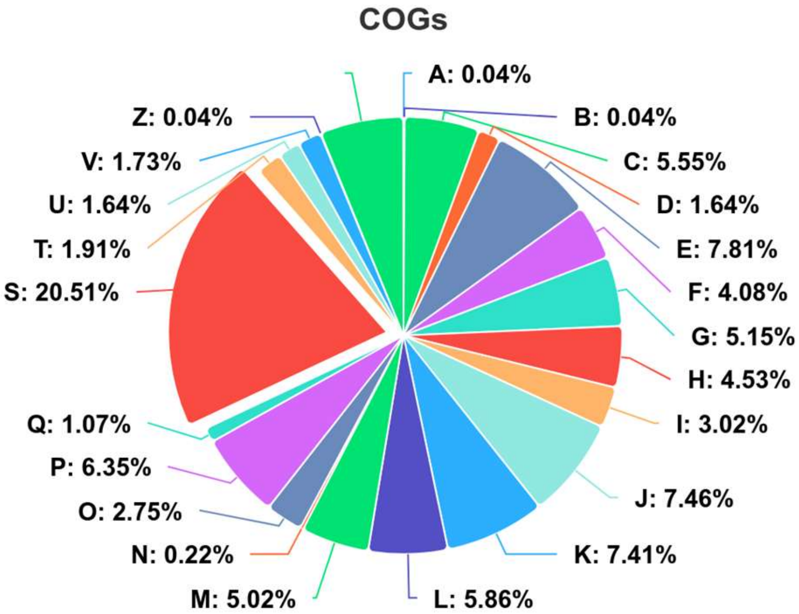

A：RNA processing and modification
B：Chromatin structure and dynamics
C：Chromatin structure and dynamics
D：Cell cycle control, cell division, chromosome partitioning
E：Amino acid transport and metabolism
F：Nucleotide transport and metabolism
G：Carbohydrate transport and metabolism
H：Coenzyme transport and metabolism
I：Lipid transport and metabolism
J：Translation, ribosomal structure and biogenesis
K：Transcription
L：Replication, recombination and repair
M：Cell wall/membrane/envelope biogenesis
N：Cell motility
O：Posttranslational modification, protein turnover, chaperones
P：Inorganic ion transport and metabolism
Q：Secondary metabolites biosynthesis, transport and catabolism
S：Function unknown
T：Signal transduction mechanisms
U：Intracellular trafficking, secretion, and vesicular transport
V：Defense mechanisms
_ ：Unclassified

Supplementary Figure. S3. Results of an eggNOG functional category analysis of strain H164^T. The major two parts of 2,114 COG categories in strains H164^T, are E (Amino acid transport and metabolism), J (Translation, ribosomal structure and biogenesis) and K (Transcription).

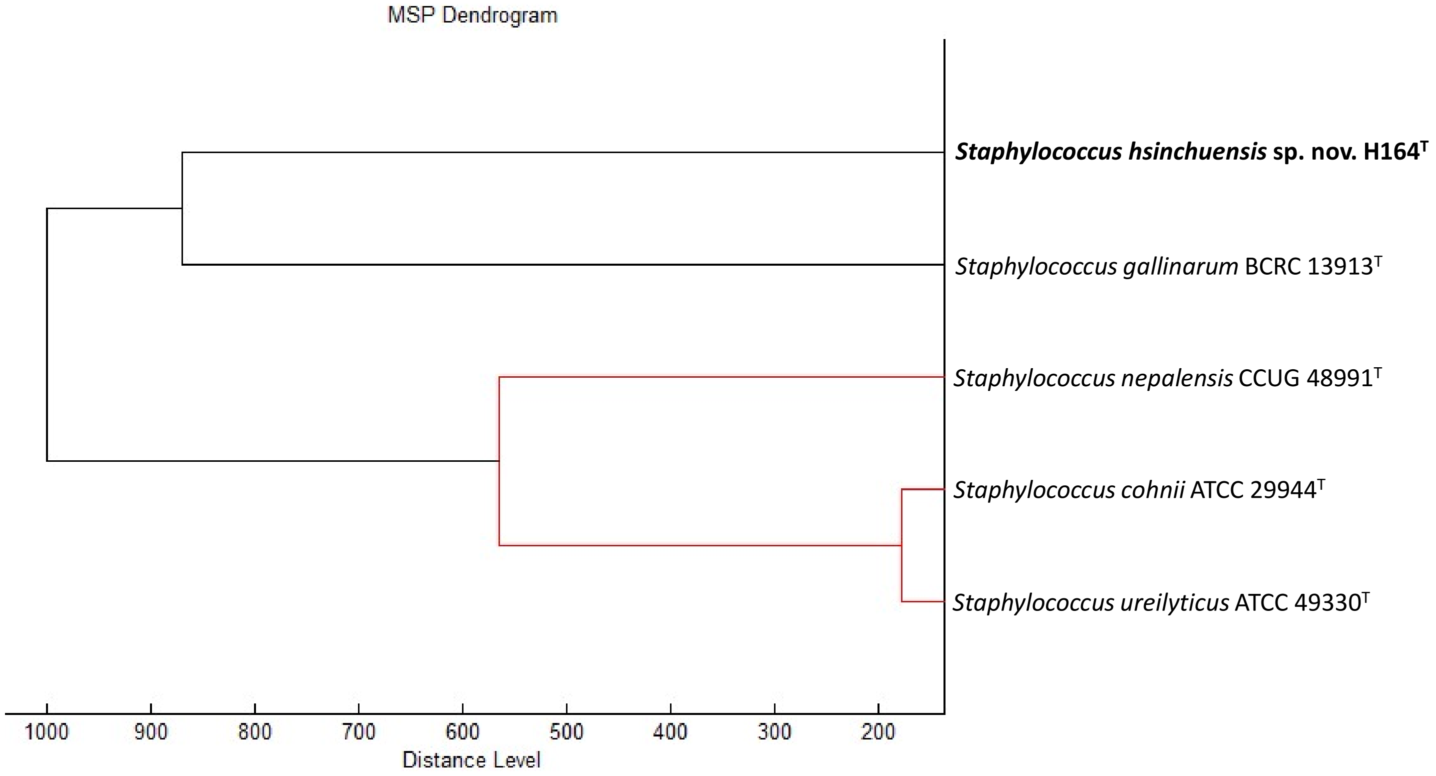

Supplementary Figure S4. Dendrogram showing the clustering of the *Staphylococcus* strains based on MALDI-TOF MS analysis.
